# Sustained impact of community-based physical activity interventions: key elements for success

**DOI:** 10.1186/1471-2458-13-892

**Published:** 2013-09-27

**Authors:** Callista Haggis, Joanie Sims-Gould, Meghan Winters, Kaitlyn Gutteridge, Heather A McKay

**Affiliations:** 1 Centre for Hip Health and Mobility, 2635 Laurel Street, Vancouver, BC V5Z 1M9, Canada; 2 Faculty of Medicine, University of British Columbia, Vancouver, BC V6T 1Z4, Canada; 3 Faculty of Health Sciences, Simon Fraser University, Burnaby, BC V5A 1S6, Canada

**Keywords:** Physical activity, Built environment, Community-based research, Social ecological model, Participatory action research, Framework, Stakeholders

## Abstract

**Background:**

Compelling evidence supports the cost effectiveness and potential impact of physical activity on chronic disease prevention and health promotion. Quality of evidence is one piece, but certainly not the sole determinant of whether public health interventions, physical activity focused or otherwise, achieve their full potential for impact. Health promotion at both population and community levels must progress beyond health intervention models that isolate individuals from social, environmental, and political systems of influence.

We offer a critical evaluation of lessons learned from two successful research initiatives to provide insights as to how health promotion research contributes to sustained impact. We highlight factors key to success including the theoretical and methodological integration of: i) a social ecological approach; ii) participatory action research (PAR) methods; and iii) an interdisciplinary team.

**Methods:**

To identify and illustrate the key elements of our success we layered an evaluation of steps taken atop a review of relevant literature.

**Results:**

In the school-based case study (Action Schools! BC), the success of our approach included early and sustained engagement with a broad cross-section of stakeholders, establishing partnerships across sectors and at different levels of government, and team members across multiple disciplines. In the neighbourhood built environment case study, the three domains guided our approach through study design and team development, and the integration of older adults’ perspectives into greenway design plans. In each case study we describe how elements of the domains serve as a guide for our work.

**Conclusion:**

To sustain and maximize the impact of community-based public health interventions we propose the integration of elements from three domains of research that acknowledge the interplay between social, environmental and poilitical systems of influence. We emphasize that a number of key factors determine whether evidence from public health interventions in school and built environment settings is applied in practice and policy sectors. These include relationship building at individual, community, and societal levels of the social ecological model, using participatory action research methods, and involving an engaged and committed interdisciplinary team.

## Background

*“Physical inactivity is the fourth leading cause of death worldwide… Although evidence for the benefits of physical activity for health has been available since the 1950s, promotion to improve the health of populations has lagged in relation to the available evidence…”*[1]

Dr. Harold W Kohl, 2012

Quality of evidence is one piece, but certainly not the sole determinant of whether public health interventions, physical activity focused or otherwise, achieve their full potential for impact [2,3]. The literature speaks to a number of other factors at play. A central consideration is that health promotion at both population and community levels demands that we progress beyond traditional health intervention models that isolate individuals from social, environmental, and political systems of influence [4-7]. In public health research, researchers must collaborate with stakeholders to generate knowledge that end-users might apply to interventions related to policy, practice, and/or product development [8-11]. Further, retrofitting interventions that are developed in isolation from the population they seek to ‘serve’ are destined to fail [6]. Importantly, the implementation of health promotion research is but a first step of an often-lengthy process toward sustained impact of effective outcomes [5,12]. We define sustained impact as the implementation of an evidence-based program or policy that has lasting influence on health promoting behaviour [5].

We offer a critical evaluation of lessons learned to provide insights on how health promotion research contributes to sustained impact. We highlight factors key to success including the theoretical and methodological integration of: i) a social ecological approach; ii) participatory action research (PAR) methods; and iii) an interdisciplinary team. Although these three domains have been incorporated in many other studies, our aim is to extend this work and explicitly illustrate how the integration of both their unique and overlapping characteristics are critical to our success in school-based-physical activity (with children and youth) and built environment (with adults over 65) settings. More generally speaking, we show that how these domains intersect or are used in concert to maximize effectiveness depends upon the problem being addressed and distinct environmental settings.

Therefore, our synopsis of lessons learned addresses a health issue of pressing importance -- physical inactivity. We focus on those elements of the three central domains that guide our approach and, illustrate it’s utility in two community-based settings: schools and the neighbourhood built environment.

### The pressing health and policy concern of physical (in) activity

The well-known benefits of physical activity and the challenge of implementing physical activity models within and across communities, sets the stage for our discussion. Compelling and incontrovertible evidence supports the cost effectiveness and potential impact of physical activity on chronic disease prevention and health promotion [13-17]. Alarmingly, physical inactivity is now cited as the fourth leading cause of death worldwide [1]. To address this pandemic, the Global Advocacy for Physical Activity group identified ‘7 best investments’, well-supported by evidence to assist populations achieve guideline levels of physical activity [18]. Two of those seven investments are: (1) promoting physical activity in school environments, and (2) neighbourhood built environments conducive to physical activity [18,19].

The issue of physical inactivity, and in particular these two ‘best investments’ serve as motivation and provide grounding context for our discussion, and its application in real-world research endeavours.

## Methods

Three researchers from our team initiated the critical evaluation that identified key elements within the domains that contributed to impact and sustainability in two large programs of research (Action Schools and Active Streets Active People). They layered an evaluation of steps taken to develop and implement the research programs atop a review of the literature. In five separate team meetings, we compared experiences and data from a process evaluation (where obtainable) with findings from a literature review. For this review we included 74 articles (Web of Science and EBSCO interdisciplinary academic databases, restricted to articles published 2000–2013) related to health intervention research impact. We isolated key elements as part of this comparison. Strategies used to establish rigor for this study included engaging with the research team for peer debriefing (via team meetings and smaller focused discussion about the developing themes), memo writing throughout the analysis process, and recording an audit trail of decisions made throughout [20-22].

The University of British Columbia (UBC) Clinical Research Ethics Board (C02-0537) approved the ethics for the Action Schools pilot study, and the Action Schools dissemination (B05-0505). The University of Victoria also approved the ethics for the Action Schools dissemination (07-05-149f). The UBC Behavioural Research Ethics Board (H12-00593) and the Simon Fraser University Research Ethics Board (2012 s0435) approved the ethics for Active Streets Active People.

## Results and discussion

### Domain 1: a social ecological approach

Societal problems, like physical inactivity, require comprehensive multi-factorial solutions [1,23,24]. Social ecological models address the interaction and interdependency between individuals, multiple settings (e.g. home, school) and levels (e.g. local government, family) that influence behaviour [25-27]. Interventions that adopt a social ecological framework identify one or more targets for change using networking relationships and rely upon partnerships across sectors and disciplines [28]. The World Health Organization (WHO) also speaks to the need of a social ecological approach to combat physical inactivity as it relates to the global burden of disease, death and disability [18]. The WHO deems it essential to engage different levels of stakeholders so that diffusion of context-specific, evidenced based findings can be integrated into institutions that influence uptake in the broader population [18]. Thus, social ecological approaches increase the potential sustainability and impact of public health research [5,26,27].

### Domain 2: a participatory action research approach

The second domain of our praxis relates to participatory action research (PAR) methodologies. PAR mobilizes community partnerships and engages stakeholders early and across phases of research to ensure opportunities to invest in research design, questions and outcomes [29]. Genuine opportunities for participation are necessary to overcome the common research pitfall of superficial levels of engagement and therefore integral to the success of many public health research projects [6]. As one example, first and foremost, researchers must engage potential users to identify relevant research questions [6]. In PAR the community identifies the “problem” to address. Research findings are directly applied (where possible) to solve these problems.

Research conducted using PAR approaches seeks to avoid traditional “extractive” methods most often adopted by universities and governments where “experts” enter a community without consultation, assess subjects and extract data from the community to write theses and publications and never report back to that community. PAR proceeds through iterative, continuous cycles where researchers and community partners work together to first identify major issues, concerns and problems, then initiate research, subsequently to originate action, and then to learn about this action; after which they proceed to a new research and action cycle. Participants continuously reflect on their learning from previous actions and proceed to initiate new actions.

Another key aspect of PAR in public health settings is early and close engagement of policy makers [30]. Political context is a significant and sometimes ‘intangible’ barrier that traditional research models do not consider. Context may determine the degree or nature of involvement, but actors from political and policy arenas should have a role in the research development and implementation process. Economic feasibility and cultivating shared values in partnership with decision makers that together translate into political will are critical for sustained impact [2,31]. Policy makers have commented upon the naivety of public health researchers regarding political processes that lead to policy action including lack of recognition that even ‘rigorously validated’ evidence requires an enabling environment to be applied in policy and practice [2,8,11]. Providing local examples with clear connections and direct impact on local community, family and constituents is key to policy makers implementing research outcomes [11].

### Domain 3: interdisciplinary teams

Convening interdisciplinary research teams is the third element we deem essential to guide the sustained impact of community-based public health interventions. The growing trend towards an interdisciplinary team approach in research addresses complex scientific and societal problems that cannot be effectively addressed within a singular disciplinary setting [32,33]. It also acknowledges that interdisciplinary teams are preferred to create a holistic, integrated view of complex phenomena [34]. Teams ideally include a cadre of trained scientists with specialized knowledge across different fields and other relevant stakeholders such as policy analysts/makers, government officials, health care workers/managers and citizens. The definition of a ‘successful’ team varies most often depending on expectations of stakeholder groups and may range from the number of publications related to the project on the one hand to the teams’ ability to translate research findings into programs, practice or policy, on the other. Given the complex conceptual, methodological and translational nature of community-based public health research we advocate an interdisciplinary approach to address key challenges that span micro, meso, and macro levels of influence [35].

### Example 1: promoting physical activity in the school environment- a focus on children and youth

Childhood obesity rates in North America continue to rise [36]. In Canada alone, research indicates that as many as 93% of children and youth may not participate in enough physical activity to maintain healthy bodies and minds [37]. Innovative strategies are necessary to encourage health-promoting behaviors, such as physical activity and healthy eating, for youth. Schools provide an important intervention environment – this setting reaches youth of diverse racial and socioeconomic backgrounds and children spend approximately 50% of their day in a classroom [38,39]. Further, evidence suggests that school-based interventions that target health behavior modification in school-aged children, including physical activity and healthy eating, are the most promising strategies to date [38].

We integrated elements from three key domains to develop our research approach and implement, evaluate and disseminate Action Schools! BC (Action Schools) -- a province-wide physical activity focused, whole school, health promotion model. The Action Schools model was built upon the premise that to ‘provide more opportunities for students to make healthy choices more often’ within elementary and middle school environments, family, school, community and provincial level support is essential. Implementation of the Action Schools model was efficacious and increased children’s physical activity, cardiovascular fitness and other health-related outcomes [40,41]. The design of the model enhanced buy-in by principals and teachers and influenced adoption of the model within schools [40]. Importantly, political will and public interest figured prominently in the sustained impact of the Action Schools model [42]. Currently, 1455 schools are registered as Action Schools, and the model has reached more than 400,000 children across BC [43,44]. This scaling-up of Action Schools was influenced by a hierarchy of factors that traversed individual, institutional and environmental levels [40].

The scope and impact of Action Schools provides one example of how elements from the three domains of our framework work in concert to inform our practice. Specifically, in-line with the principles of PAR, we had early and sustained engagement with a broad cross-section of stakeholders to address physical inactivity in a young population. In the pilot phase, we created a research partnership with five government agencies to address the role of two-way knowledge exchange in uptake and application of innovations (Table 1) [45]. Also, taking from the social ecological approach we formed partnerships horizontally across sectors and vertically at different levels of governance by creating three guiding committees (1) Provincial Advisory Committee (2) School Advisory Committee (Action Team) (3) Evaluation Committee [41]. Combining these elements, we maintained ongoing engagement during the research and implementation process through a series of consultations and focus groups. For example, we hosted meetings with principals and administrators to learn the pressing health issues in their school environment. We also engaged teachers to overcome implementation barriers, and develop collaborative strategies to encourage students to be more active and healthy. We conducted focus groups with parents to understand their perceptions about the importance of physical activity and the impact of Action Schools on their child’s life. We provided baseline pilot data to the guiding committees within 4 months of the beginning of evaluation, immediate process evaluation data to program developers and actively involved stakeholders in the dissemination of findings and plans for program sustainability.

**Table 1:** Action schools: multi-level model of engagement

**Individual level**	**Elementary and middle school-aged children**
Community level*	Elementary and middle school principals
	Elementary and middle school teachers
	Parent Advisory Council (PAC)
	District school board superintendents
	Post-secondary institutions (including University of British Columbia, University of Victoria)
	Sport and leisure governing bodies
	Aboriginal sport, recreation and physical activity councils
	Community-based health promotion organizations (including BC Paediatric Society, Heart and Stroke Foundation, Childhood Obesity Foundation)
	School-based healthy living committees (Breakfast for Learning, BCRPA After School Initiative)
	Active transportation initiatives (including HASTe BC, Move for Health Day)
	Interdisciplinary working groups (including Physical Activity and Obesity Working Group, Physical Literacy Working Group)
	Municipal governments (including City of Vancouver, City of Burnaby)
	Vancouver Board of Parks and Recreation
**Societal level**	BC Provincial Health Services Authority
	BC Ministry of Health (Healthy Families BC)
	BC Ministry of Community, Sport and Cultural Development
	BC Ministry of Education (Healthy Schools BC)
	2010 Legacies Now
	Directorate of Agencies for School Health BC (DASH BC)
	Public Health Agency of Canada (Aboriginal Diabetes Initiative)

*For complete list of current Action Schools stakeholders, please refer to Action Schools! BC report [43].

Consistent with principles of PAR, our guiding committees transformed with the scale-up of Action Schools to integrate more diverse interests and stakeholders. Currently, over 50 organizations, initiative or committees 2011–2012 are engaged with Action Schools including Heart and Stroke Foundation (BC and Yukon), Childhood Obesity Foundation, BC Paediatric Society (Table 1) [43].

Our interdisciplinary team integrated committed scientists from 6 disciplines to design and implement Action Schools. Research team members spanned education, medicine, kinesiology, cardiovascular physiology, psychology and health policy. This interdisciplinary research team complemented the needs and expertise of stakeholders and was positioned to integrate stakeholder perspectives and insights into research questions and methodologies specific to investigators’ specialized area of enquiry (e.g. cardiovascular health, psychosocial health, bone health and so on).

### Example 2: the intersection of physical activity and the neighbourhood built environment- a focus on older adults

The built environment, that being the physical form of neighbourhoods including land use patterns, street-scape features and transportation systems, has the potential to significantly shape health outcomes, positively and negatively [46-48]. The WHO positions healthy urban planning as a top priority to improve health globally [49,50]. Researchers and practitioners identify a need to realign the built environment with public health to overcome the weak dialogue and disconnections that currently characterize the relationship between the disciplines of urban health and planning [51,52]. Further, the National Centre for Environmental Health of the Centres for Disease Control and Prevention argues for the “reintegration of land use planning and public health, explicitly linking transportation and land use planning to public health outcomes such as increased obesity, asthma and mental health” [51].

Importantly, engagement across multiple levels of government and community are important determinants of whether evidence based recommendations for health promotion at the built environment level are applied in practice and policy environments [51-54]. Taken together, the complexity of these issues suggests that health interventions that involve urban planning, the built environment and physical activity may benefit from an integrated approach.

Our research program in this arena, “Active Streets, Active People”, is predicated upon the notion that neighbourhood design mediates the decline in health and physical activity that accompany ageing. A walkable neighbourhood may delay the often traumatic transition of older adults to residential care or assisted living facilities and the substantial health care costs that ensue [55]. The aim of Active Streets, Active People is to identify links between the built environment and older adult physical activity and health [56,57]. The study capitalizes on a natural experiment, where major investments (> $5 Million Cdn) are being made by the City of Vancouver to develop a greenway to encourage physical activity through active transport routes and destination place making [58].

To evaluate the impact of the greenway on older adults’ physical activity and social interactions, we recruited 193 older adults who live in surrounding neighbourhoods to participate in a mixed-methods study. A subset of participants completed a semi-structured, qualitative walking interview. We measured participants before the greenway development (Fall 2012) and will meet with individuals again upon completion of the greenway (Fall 2014).

An integration of elements from a social-ecological model, PAR, and an interdisciplinary team approach guided both development of the Active Streets, Active People research program and the integration inclusion of older adults’ perspectives into City of Vancouver greenway design plans. Adopting central tenets of the social ecological model, we engaged partners at multiple levels and across sectors to identify feasible and high priority investments in the built environment on a neighbourhood scale (Table 2). Specifically, we convened representatives from the BC Ministry of Health, provincial health authorities, City of Vancouver (Planning, Social Policy and Engineering), seniors’ organizations and older adults themselves at a very early stage (Table 2).

**Table 2:** Active streets active people: social ecological model of engagement

**Individual level**	**Community dwelling older adults**
**Community level**	South Vancouver Neighbourhood House
	Seniors Advisory Committee, City of Vancouver
	West End Seniors’ Network
	United Way of the Lower Mainland (Strategic Initiatives (Seniors), Community Impact and & Investment)
	City of Vancouver (Planning, Engineering, Social Policy)
	City of Surrey (City councillor)
	Union of BC Municipalities (Policy Analyst)
**Societal level**	BC Housing (VP Operations)
	Vancouver Coastal Health Authority
	Ministry of Health
	Canadian Urban Institute
	World Health Organization

In keeping with PAR principles, and very early on in the research process, we hosted a day long symposium where 140 stakeholders across multiple sectors came together to address the following question(s) “What makes a neighbourhood a good place to grow old?” “What programs and services are currently available to keep older adults active?” and “How can we do better?” Additionally, our research team guided neighbourhood-walking tours with our stakeholders (older adult residents, representatives from community-based organizations, the media and policy makers). We used a street audit tool to discuss specific features of the built environment with older adults and to identify what helps or hinders their ability to be physically active in their neighbourhood. Input from the symposium and tour informed specific research questions and the study design for research activities. In addition, institutional partners from the City of Vancouver and collaborators from community-based organizations were consulted to provide feedback on the scope of the quantitative measurement toolkit and walking interview guide.

Overlapping elements of these domains also informed our strategy of ongoing engagement. One example was the distribution of the walking tour findings report (written in accessible language) to our broader community of stakeholders (Table 2). Further on in the research process, to connect stakeholders with outcomes, we provided baseline results and additional supplementary reports for those who expressed interest in specific outcomes. Participant feedback reports were distributed within 3 months of beginning the evaluation. Overall, this early and sustained engagement created ‘joint’ ownership of problems *and* solutions related to the intersection between the built environment and the health and physical activity of older adults.

As the Active Streets, Active People research program progresses into its second year we have regular (weekly to bi-monthly depending on activities) meetings with stakeholders to discuss a range of issues. Topics include: progress to date; feedback on research questions and participant feedback reports; and the design of future community-based engagement events targeted at older adult participants. Thus, in keeping with our praxis, older adults, municipal and non-governmental organization partners helped define the problem addressed, methods used (quantitative and qualitative) to assess the problem and will engage with us to interpret and disseminate our results at study completion (2014).

An interdisciplinary team is of particular importance when working at the confluence of the broad fields of public health and urban planning - specifically when addressing how environmental changes affect physical activity and mobility patterns. These two disciplines have traditionally focused on different strategies to address similar issues [59]. However, the importance of interventions that address i.) factors that influence health on individual, interpersonal, and societal levels and ii.) the interplay of built environment features, lends itself to the formulation of an interdisciplinary team. Our Active Streets, Active People team is comprised of researchers with backgrounds in: Public Health, Epidemiology, Urban Planning, Gerentology, and Exercise Science. Additionaly, a senior member from the Active Transportation Division in the Department of Engineering Services at the City of Vancouver is a Co-Principal Investigator. Collaboaration, where different perspectives are valued, is key to playing to team members strength and overcoming tradtional pitfalls of narrow metrics and shortsightedness.

Clearly, there is momentum across multiple levels that call for integration between evidence-based health outcomes and policy and practices within the built environment setting. The Canadian Institute of Planners advocates for interdisciplinary partnerships that position *Healthy Communities* “as a dominant public policy and research focus on the Canadian landscape” [60]. Closer to home, the City of Vancouver developed a *Healthy City Strategy* that spans municipal departments and includes events that facilitate interdisciplinary dialogue between health researchers and practitioners [61]. To illustrate the mutual benefit received through our close relationship with urban planners and our teams’ ability (through ongoing meetings with interdisciplinary team members) to translate research and make it applicable to audiences beyond applied scientists, we were the only academic team invited to present our research at Healthy People, Healthy City Conference, a high profile event with city planners and policy makers. The integration of elements from the socio-ecological model, PAR and an interdisciplinary team approach has significantly strengthened our research design, engagement with stakeholders and our ability to translate new knowledge into meaningful outcomes for stakeholders so as to ‘scale-up’ or disseminate these outcomes, in future.

## Conclusions

Wicked problems – defined as challenging issues with diverse, changing, and context specific factors of influence [62] such as physical inactivity do not have a single, or simple solution [63]. Complexity requires an advancement of processes, tools and teams that are attuned to these conditions [64]. Specifically, to sustain and maximize the impact of community-based public health interventions in school and neighbourhood environment settings we propose a praxis that acknowledges the interplay between social, environmental and political systems of influence. We join others who have advanced beyond interventions that target individuals and small groups isolated from their context [2]. We emphasize that key factors for success are relationship building at individual, community, and societal levels of the social ecological model, using participatory action research methods, and the involvement of an engaged and committed interdisciplinary team. The view supported by our work is that empowerment at multiple levels seeks to ignite changes that, in turn, contribute to sustained impact of outcomes [27]. We also advocate for strong and early alliances with government and community stakeholders. These collaborators informed our process at every stage and become key dissemination partners and generate evidence-informed policies, in future.

## Competing interests

The authors declare that there are no competing interests.

## Authors’ contributions

The conceptualization and development of this critical evaluation was led by CH and JSG. CH led the literature review and writing of the paper. KG assisted with the literature review and wrote portions of the manuscript. CH, JSG, MW, and HM refined the critical evaluation and wrote significant portions of the manuscript. All authors critically revised the manuscript and approved the final version.

## Pre-publication history

The pre-publication history for this paper can be accessed here:

http://www.biomedcentral.com/1471-2458/13/892/prepub
